# Lightning performance analysis of a rooftop grid-connected solar photovoltaic without external lightning protection system

**DOI:** 10.1371/journal.pone.0219326

**Published:** 2019-07-11

**Authors:** M. S. M. Nasir, M. Z. A. Ab-Kadir, M. A. M. Radzi, M. Izadi, N. I. Ahmad, N. H. Zaini

**Affiliations:** 1 Centre for Electromagnetic and Lightning Protection Research (CELP), Advanced Lightning, Power and Energy Research Centre, Faculty of Engineering, Universiti Putra Malaysia, UPM, Serdang, Selangor, Malaysia; 2 Faculty of Engineering, Universiti Pertahanan Nasional Malaysia, UPNM, Kem Sungai Besi, Kuala Lumpur, Malaysia; 3 Institute of Power Engineering, Universiti Tenaga Nasional, Kajang, Selangor, Malaysia; 4 Centre for Advanced Power and Energy Research (CAPER), Advanced Lightning, Power and Energy Research Centre, Faculty of Engineering, Universiti Putra Malaysia, UPM, Serdang, Selangor, Malaysia; University of Science and Technology Beijing, CHINA

## Abstract

The Sustainable Energy Development Authority of Malaysia (SEDA) regularly receives complaints about damaged components and distribution boards of PV systems due to lightning strikes. Permanent and momentary interruptions of distribution circuits may also occur from the disturbance. In this paper, a solar PV Rooftop system (3.91 kWp) provided by SEDA was modelled in the PSCAD/EMTDC. The Heidler function was used as a lightning current waveform model to analyse the transient current and voltage at two different points susceptible to the influence of lightning events such as different lightning current wave shape, standard lightning current and non-standard lightning current. This study examines the effect on the system components when lightning directly strikes at two different points of the installation. The two points lie between the inverter and the solar PV array and between inverter and grid. Exceptionally high current and voltage due to the direct lightning strike on a certain point of a PV Rooftop system was also studied. The result of this case study is observed with and without the inclusion of surge protective devices (SPDs). The parameters used were 31 kA of peak current, 10 metres cable length and lightning impulse current wave shape of 8/20μs. The high current and voltage at P1 striking point were 31 kA and 2397 kV, respectively. As for the AC part, the current and voltage values were found to be 5.97 kA and 5392 kV, respectively.Therefore, SPDs with suitable rating provided by SEDA were deployed. Results showed that high transient current voltage is expected to clamp sharply at the values of 1.915 kV and 0 A at the P1 striking point. As for the AC part, the current and voltage values were found to be 0 kA and 0.751 V, respectively. Varying lightning impulse current wave shapes at striking point P2 showed that the highest voltage was obtained at waveshape 10/350 μs at 11277 kV followed by wave shapes of 2/70 μs, 8/20 μs and 0.7/6 μs. The high value of transient voltage was clamped at a lower level of 2.029 kV. Different lightning amplitudes were also applied, ranging from 2–200 kA selected based on the CIGRE distribution. It showed that the current and voltage at P1 and P2 were directly proportional. Therefore, the SPD will be designed at an acceptable rating and proper position of SPD installation at solar PV Rooftop will be proposed. The results obtained in this study can then be utilised to appropriately assign a SPD to protect the PV systems that are connected to the grid. Installing SPDs without considering the needs of lightning protection zones would expose the expensive equipment to potential damage even though the proper energy coordination of SPDs is in place. As such, the simulation results provide a basis for controlling the impacts of direct lightning strikes on electrical equipment and power grids and thus justify SPD coordination to ensure the reliability of the system.

## Introduction

Rapid growth in energy developments and demands for renewable energy (RE) show that the implementation of renewable energy is vastly expanding. Compared to the other types of renewable energy, solar energy is prominent, as an infinite resource, natural, ecological, friendly, and economical [1]. The potential availability of solar energy is significantly greater than the current overall global energy demands. Solar energy has been developing more rapidly than the other RE sources for a few decades now. The photovoltaic (PV) systems are employed to convert the power of the sun from sunlight energy to electrical energy [1]. In the future, solar power generation will be crucial for a sustainable form of energy. Moreover, solar irradiation is generally in abundance so that the electricity demands of the world can be met to a large extent by solar power technologies alone. The experts predict that by the year 2050, renewable energy could be generating over 50% of all the supply and 80% of all electricity system would come from it [2]. No surprisingly, the Malaysian government is interested and committed to the development of solar energy in the country as one of the important sources of energy [3].

The Sustainable Energy Development Authority Malaysia (SEDA) is a statutory body that was formed under the Sustainable Energy Development Authority Act 2011. This body was formed to ensure the effective growth of the RE sector in Malaysia. SEDA has been tasked with the responsibility to manage the implementation of Fit-in tariff (Fit) mechanism. Moreover, SEDA must ensure that sustainable energy is managed properly and plays an important role in the development of the nation’s economy. The plan of FiT initiated by the Malaysian government under National Renewable Energy Policy and Action Plan (2010) facilitates the efficiency of RE contribution and funds, besides ensuring the growth of the RE industry. Therefore, the Renewable Energy Act 2011 was gazette on 2^nd^ June 2011 published for this purpose [4].

Lightning is a major issue faced by Tenaga Nasional Berhad (TNB) in Malaysia. Statistically in Malaysia, lightning causes over 70% of power failures. In Germany, statistical data show that 26% of the damages are caused by lightning. It may cause permanent or momentary interruptions on distribution circuits. With the rapid increase of sensitive loads, momentary interruptions are not acceptable and are a serious issue that has to be addressed. Lightning can cause damage or malfunctioning of the electrical, communication or automation systems that can cost more than 250 million [4,5].

Photovoltaic (PV) systems are normally installed in wide open outdoor places such as on the rooftop or a solar farm. This leaves the electrical or electronic equipment exposed to lightning strikes nearby. The operation of the electrical or electronic equipment power system placed outside or inside the building can be interrupted with economic implications to the power system operation. Hence, a complete lightning protection system in a PV installation is very crucial and a practical requirement to avoid the interruption of the system, while destruction as well as faults leading to casualties will also be avoided [2,6].

Direct and indirect lightning strikes have great potential in affecting the whole of a PV Rooftop system. The nature of its installation on rooftops easily exposes s the panels to a direct hit. The situation is made even worse if the installation is made in a high lightning density area. This will result in malfunction or destruction of the PV Rooftop system if it is drastically affected by lightning. Direct strikes can destroy PV panels, inverters, cables, and fuses due to the high current. On the other hand, indirect strikes would induce high voltages into the system and consequently, the conductors, PV panels and other components would be affected. This will eventually emit sparks that could cause fire and explosion to the inflammable materials of the system.

A sensitivity analysis is necessary for the development of lightning overvoltage in a Rooftop PV system, bearing in mind the impact of lightning striking spot, the lightning current amplitude, the building height, the soil resistivity and the distance between the solar arrays and the external protection system. The PV Rooftop system is commonly located in high-rise buildings which makes it very prone to lightning strikes [7].As far as Malaysia is concerned, no standards exist on lightning protection for PV systems, except for MS 1837:2010 which focuses on the PV installation. Thus; there were no previous studies that dealt with lightning surge analysis prior to the solar PV installation for residential and commercial buildings or solar farms.

There is no circuit model or test for the PV Rooftop system dedicated to lightning surge studies, especially in aspects of SPD placement, selection of suitable ratings, cable length, and sizing, and number of SPDs required. Direct strikes may trigger fires and even explosions to the PV Rooftop installation. In the case of indirect strikes, induced overvoltages may result in outages of the electrical and electronic components inside the building.

Most PV Rooftop systems installations are not properly or adequately protected from lightning. especially when it comes to SPD installation, where no mandatory requirements are imposed. Thus, many concerns are raised on the safety and protection of the inverter. On the other hand, PV Rooftop systems may suffer from severe damage that comes from failure of the electrical and electronic parts in a PV Rooftop system, interrupting their normal operational functionality. Therefore, it is clear that lightning protection system installation is crucial in determining the life span of a PV Rooftop system [6].

According to Naxakis et al.[8], the single-crystalline silicon PV module was tested under lightning impulse voltage to evaluate its performance. It was found that PV module was completely damaged and electrically degraded when testing under lightning impulse voltage up to 144 kV. Then, tests according to IEC 61730–2 for 12 kV and up to 35 kV, PV module did not show any indication of mechanical damage and no obvious electrical degradation.

Based on the work of Jiang and Gryzbowski [9], it was found that highest possible power output of PV module would degrade exponentially with lightning impulse voltages. There are five testing voltage levels which are: 15 V, 30 V, 90 V, 400 V and 1000 V. Even with low level of lightning impulse voltage, the results showed the PV module was electrically degraded but did not have any abnormal damage even for 1000V.

Previous work by Sekioka [10] indicated that direct lightning strike will cause surface discharge to occur in grounded frame PV panel and recommended that the protection for PV panel should be considered.

Both Belik [11] and Abdul Rahim et al. [12] highlighted that the induced voltage occurrence was due to indirect lightning and caused a high voltage spark between the cables and PV modules and thus severely damaging the PV panel. The extent of damage is proportional to the distance of the spark discharge from the PV panels where the closer the distance between PV panel and spark discharge, the more severe the damage caused by the induced overvoltage.

This paper focuses on lightning surge analysis to rooftop solar PV installation under direct strike at two different locations, taking into account the variation of current waveforms (both standard and non-standard waveforms). However, this paper will only considered the lightning surge analysis on the rooftop installation without any external lightning protection and without considering any protection devices.

## Methodology

### Solar PV array modelling

**Fig 1:**
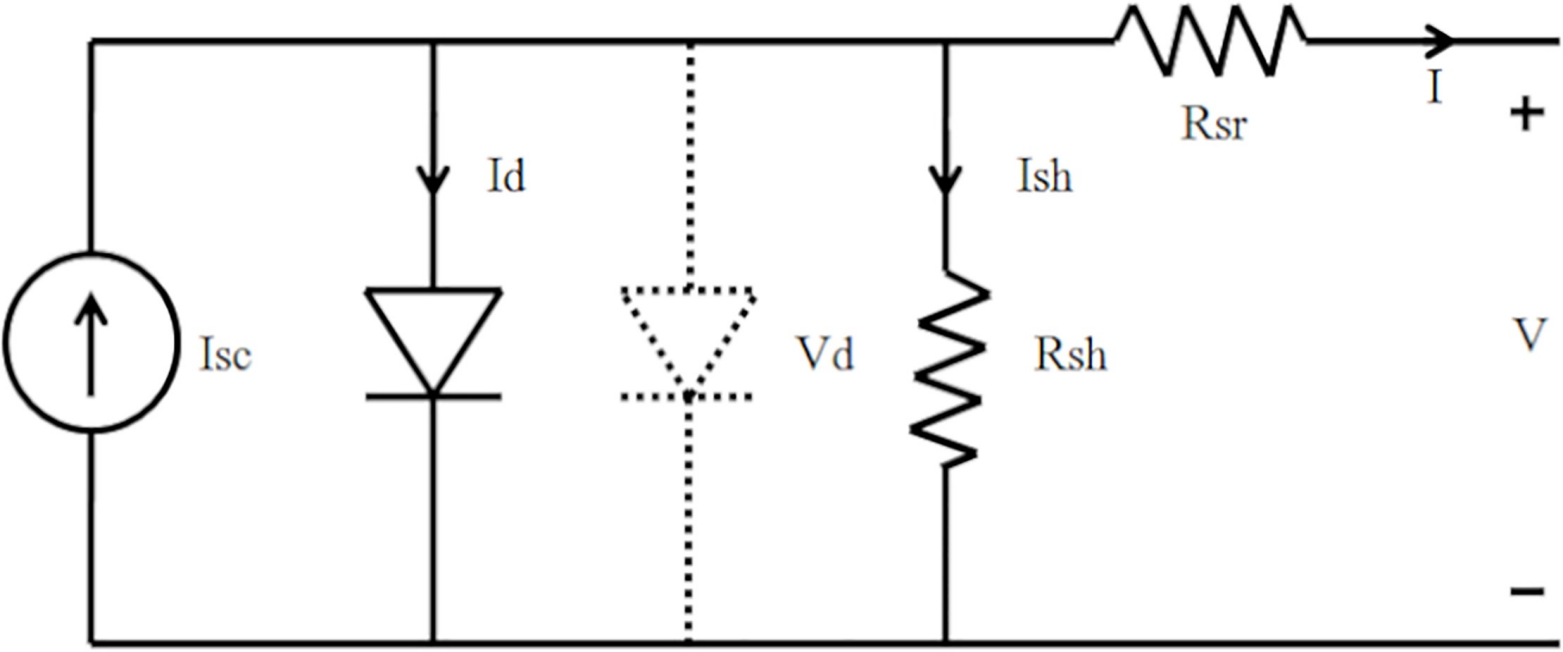
PV cell equivalent circuit.

Fig 1 shows an equivalent circuit model of single PV cell which consists of output voltage, V and the current of the PV cell, I [13]. Additionally, a current source anti-parallel with a diode, a shunt resistance, Rsh and a series resistance, Rsr as shown in Fig 1 is present with the PV Cell. Furthermore, to produce the nonlinear I-V characteristics of the PV cell, current,I_d_ that flows through the anti-parallel diode plays an important role, [13,14].

### Maximum Power Point Tracking (MPPT)

The MPPT algorithm is used to track the operating point to get the maximum power [15]. MPPT algorithm is required for tracking the maximum power point due to the continuous change of ambient temperature and irradiance of the solar source. There are two well-known MPPT algorithms which are: perturb and observe method, and incremental conductance method, [16,17].

### Inverter single-phase modelling

**Fig 2:**
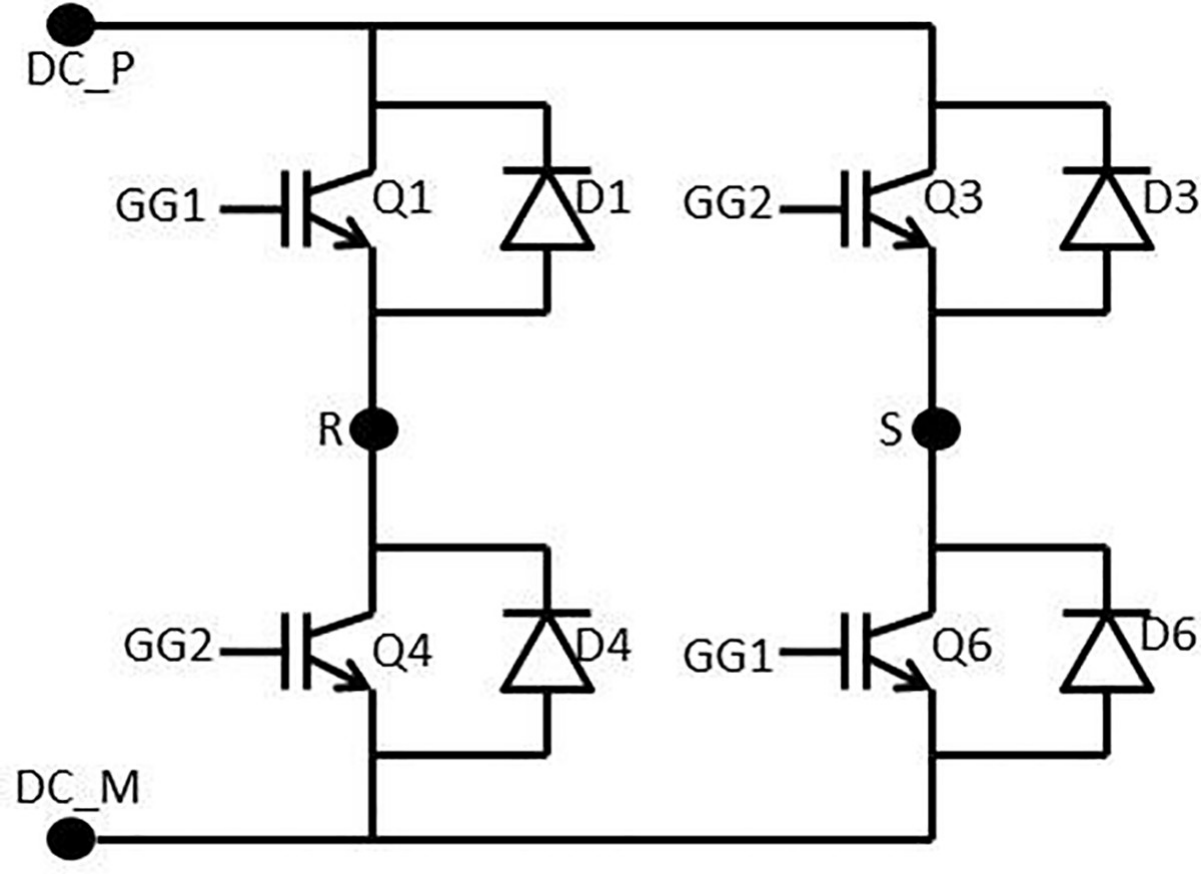
Single-phase H-bridge inverter.

The inverter is an interface of the DC source to the grid. As shown in Fig 2, there is a single phase H-bridge inverter in this study. H-bridge inverter was chosen for its efficiency [18].

**Fig 3:**
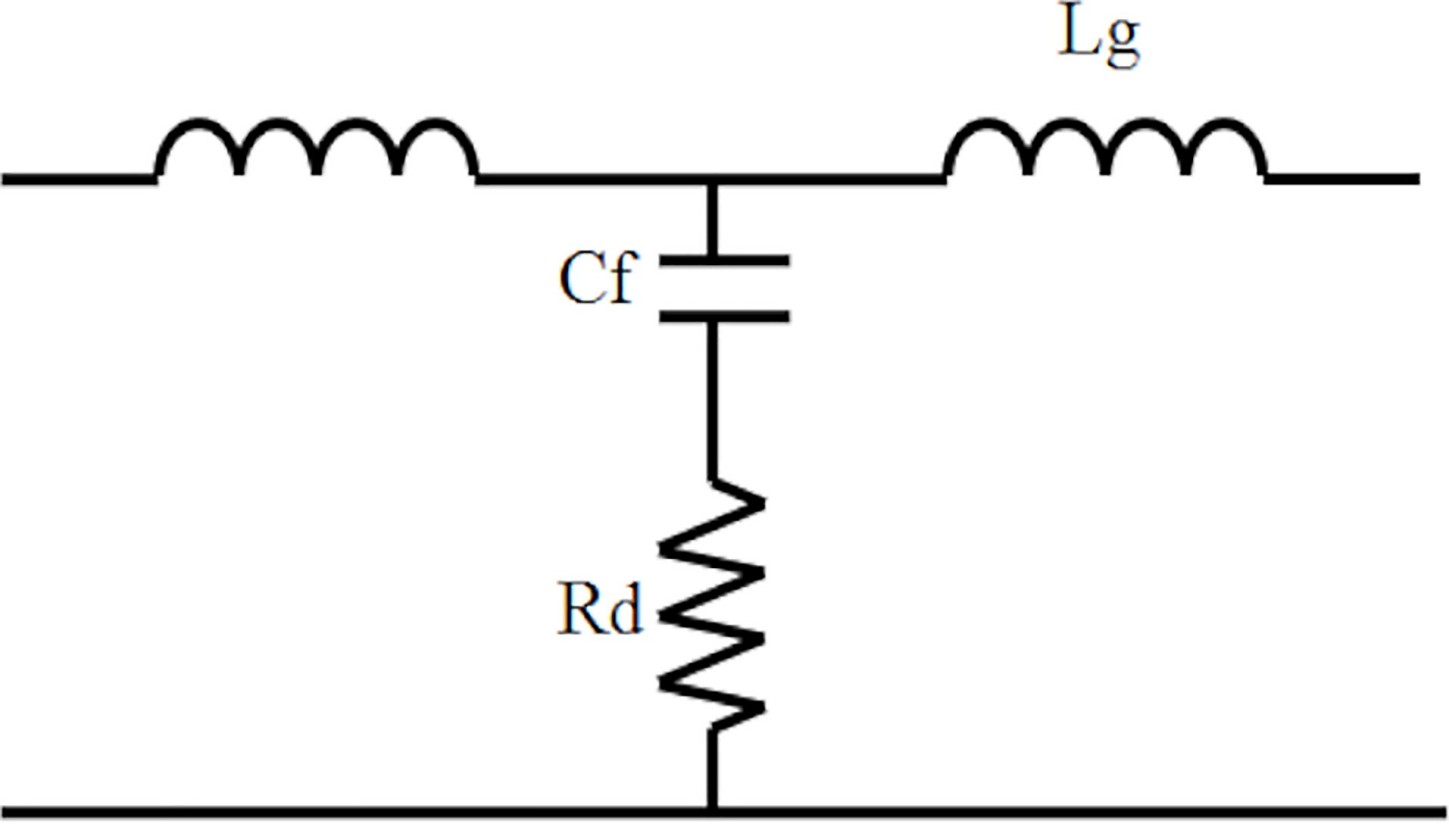
LCL filter model.

LCL filter is the main interface between the power grid and the inverter in the grid connected [19].Fig 3 shows the LCL filter that must be modelled properly to avoid distortions. Its function must also be enhanced to reduce harmonics on the output [20].

### Cable configuration

Cable configuration and modelling were carried out in PSCAD/EMTDC. As per manufacturer’s datasheet, the PV Rooftop uses cable sizes of 4 mm^2^at the DC part and 10 mm^2^ at the AC part, respectively.

### Lightning current model

There are several mathematical expressions that previous researchers have used to represent lightning current waveforms in lightning protection study. The two mathematical expressions for lightning current wave shapes are Heidler function and Double exponential that are frequently used in the lightning protection study. The double exponential shown in Eq 1 was applied to calculate *i(t)*, with *I*_*p*_ is as the peak current; and α and β are formula constants for obtaining lightning current waveform, [21, 22].

$$i(t)=I_{p}[e^{-\alpha t}-e^{-\beta t}]$$ (1)

The Heidler function that was used in this study is frequently used in several standards and defined in Eqs 2 and 3[21]:

$$i\;(t)=\frac{I_{p}}{\eta }\frac{{(t/\tau_{1})}^{n}}{1+{(t/\tau_{1})}^{n}}exp\;\left(-\frac{t}{\tau_{2}}\right)$$ (2)

where

**Fig 4:**
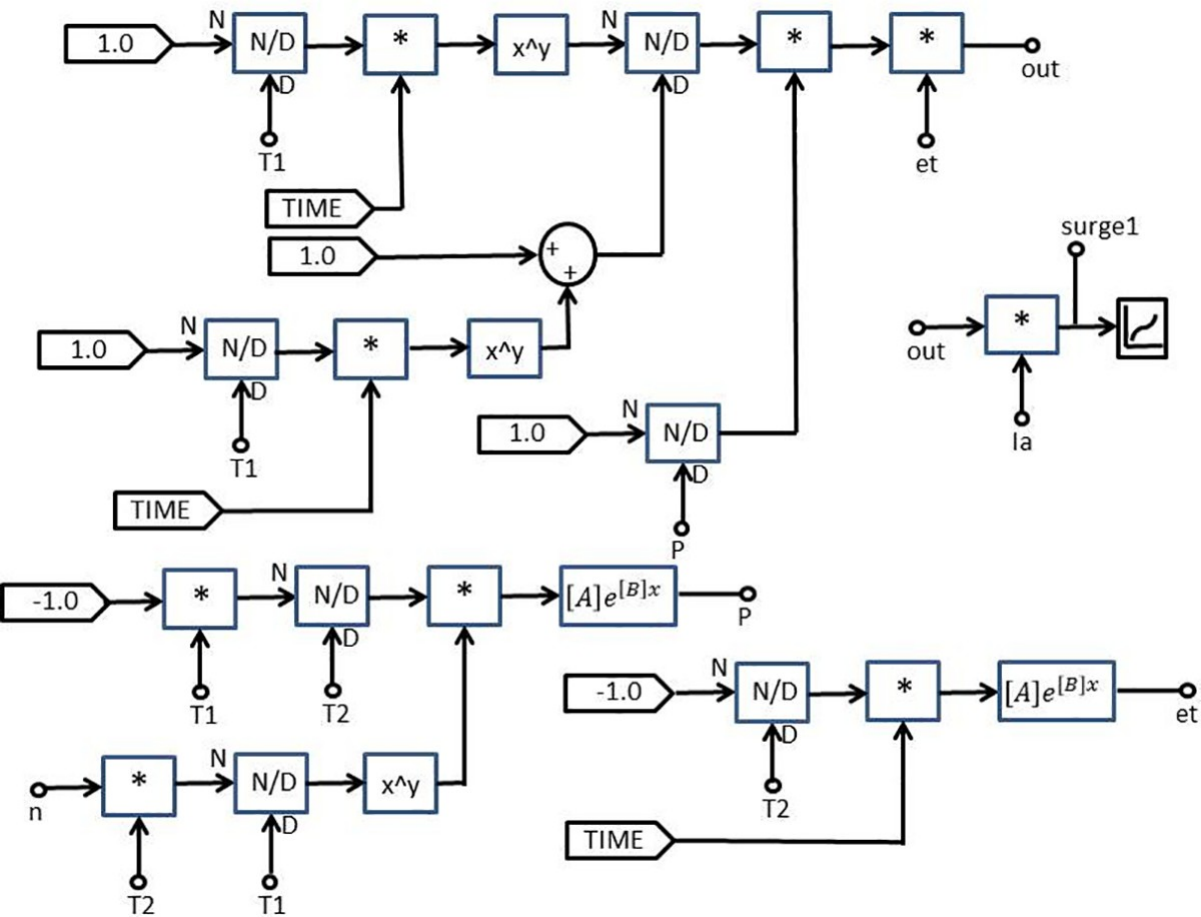
Modelling of lightning current wave shape (Heidler function).

η=exp[−τ1τ2][(nτ2τ1)(1n+1)] (3)

with *Ip* is the maximum current value, *η* is the peak current correction factor, *τ*_1_ and *τ*_2_ are the time constants to determine current rising and current decaying time, respectively and also the maximum of the current steepness. The Heidler function is used to model the lightning current waveshapes shown in Fig 4 [23].

The design and model of the PSCAD of the four lightning current wave shapes based on the Heidler function are according to the standard current wave shapes (10/350 μs and 8/20μs) and non-standard wave shape (0.7/6 μs and 2/70 μs). Lightning impulse current wave shapes with different front time and tail time are applied to determine the effect of voltage and current. For standard current wave shape (10/350 μs), it has longer tail time. Then for non-standard current wave shape (0.7/6 μs), it has short front time and tail time but for current wave shape (2/70 μs), it has long tail time. From Table 1 shows the parameters of the lightning current wave shape that was modelled in the PSCAD.

**Table 1:** Lightning current wave shape parameters.

Lightning Current Wave shapes, μs	*τ*_1_, μs	*τ*_2_, μs	*n*
8/20	5.9	11.645	2
10/350	1	475	2
0.7/6	0.177	7	2
2/70	0.28	95	2

**Fig 5:**
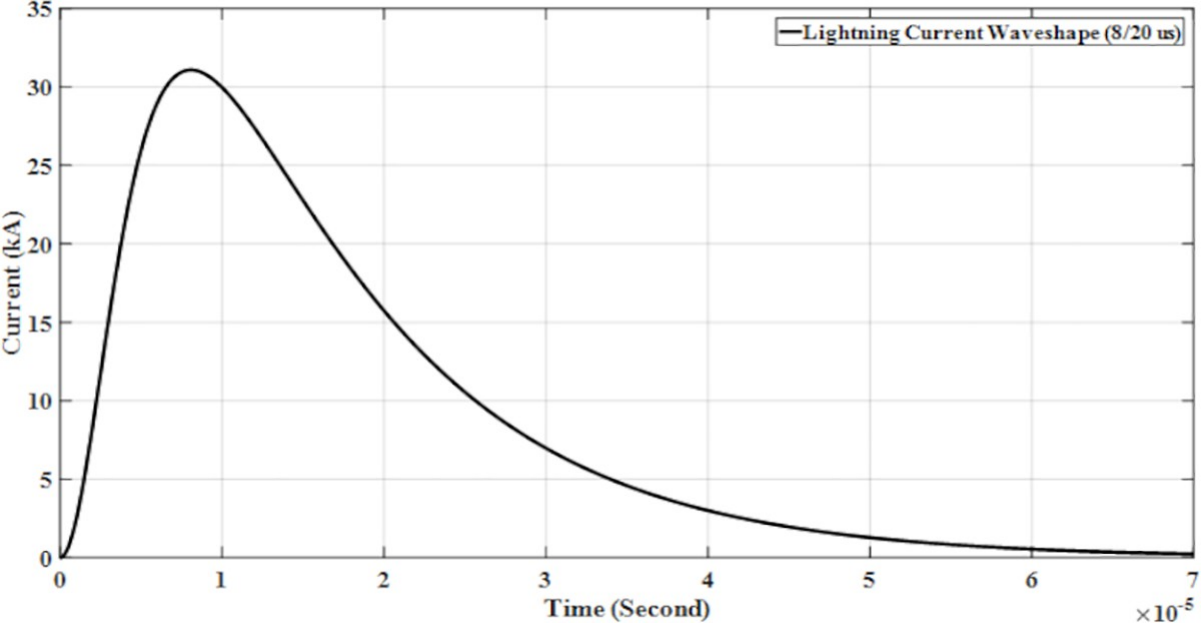
Lightning current wave shape (8/20 μs).

**Fig 6:**
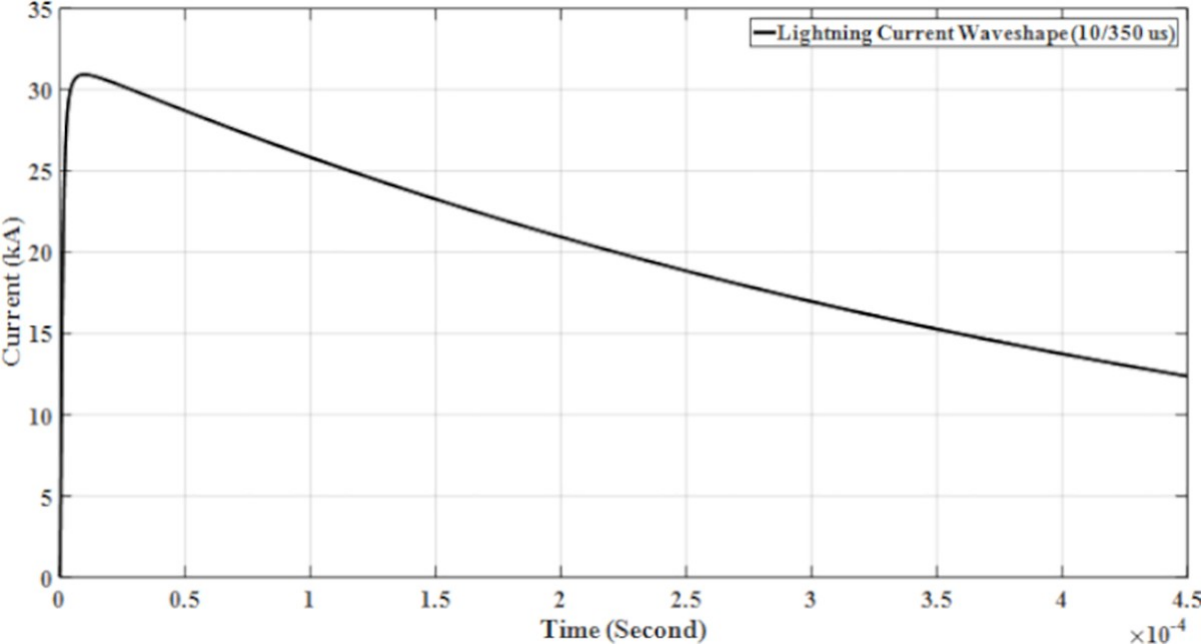
Lightning current wave shape (10/350 μs).

**Fig 7:**
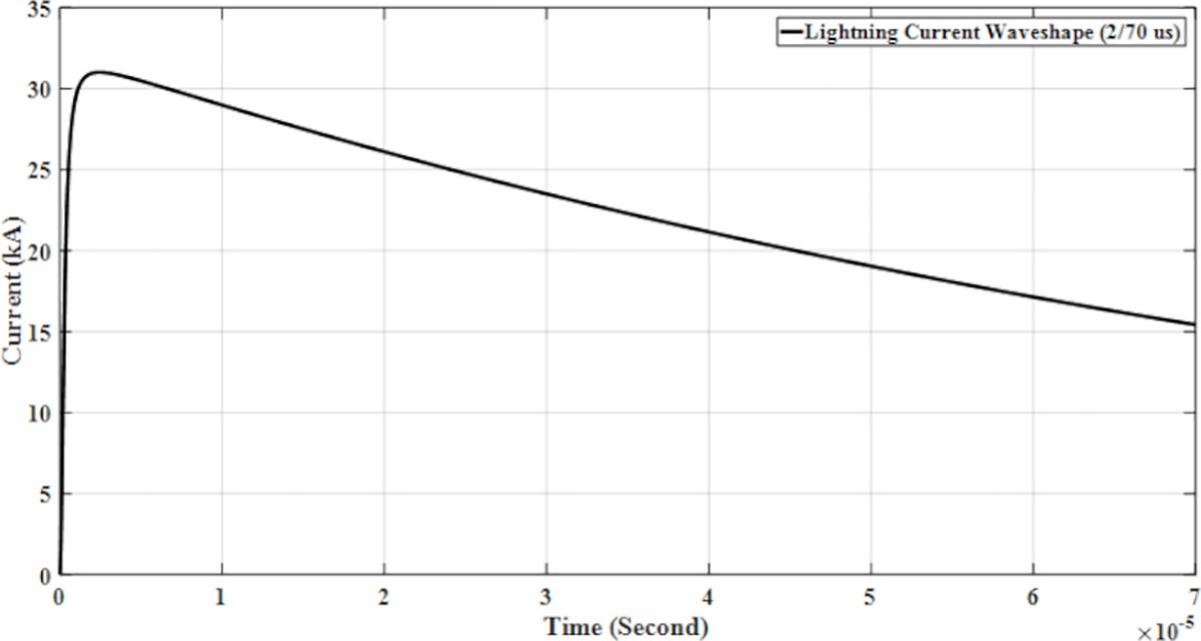
Lightning current wave shape (2/70μs).

**Fig 8:**
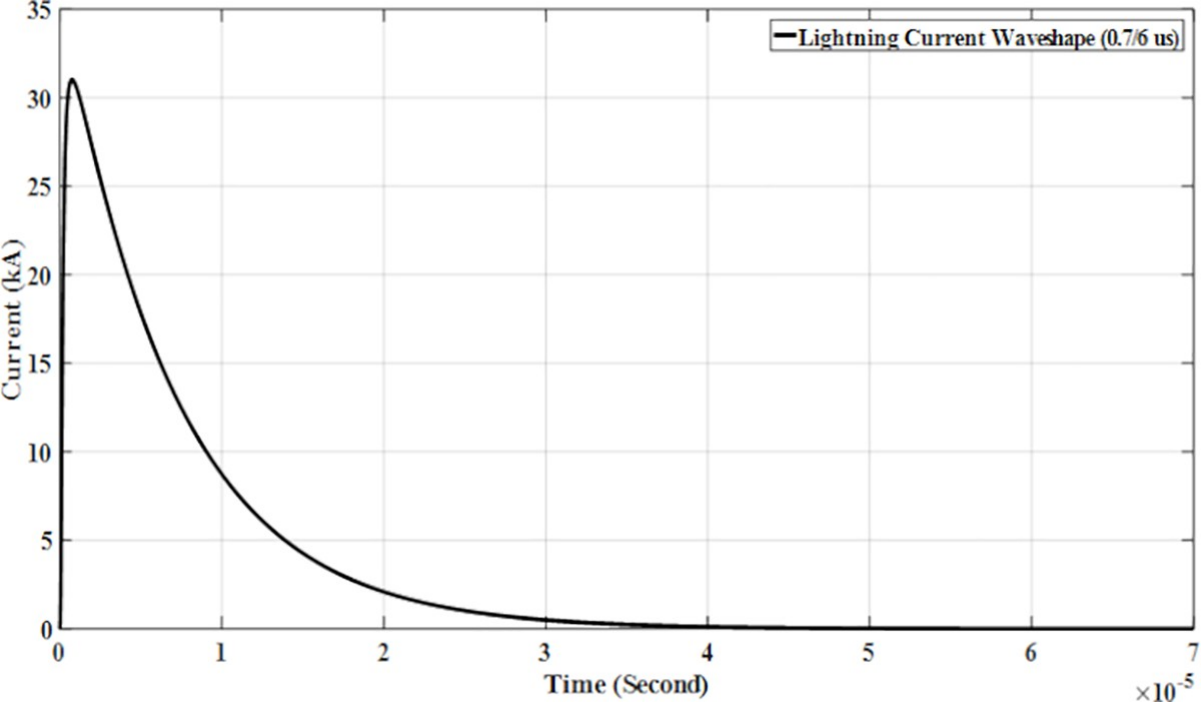
Lightning current wave shape (0.7/6μs).

The parameters for lightning current wave shape modelled in the PSCAD are shown in Table 1 and presented in Figs 5–8.

### Validation for solar PV rooftop system

**Fig 9:**
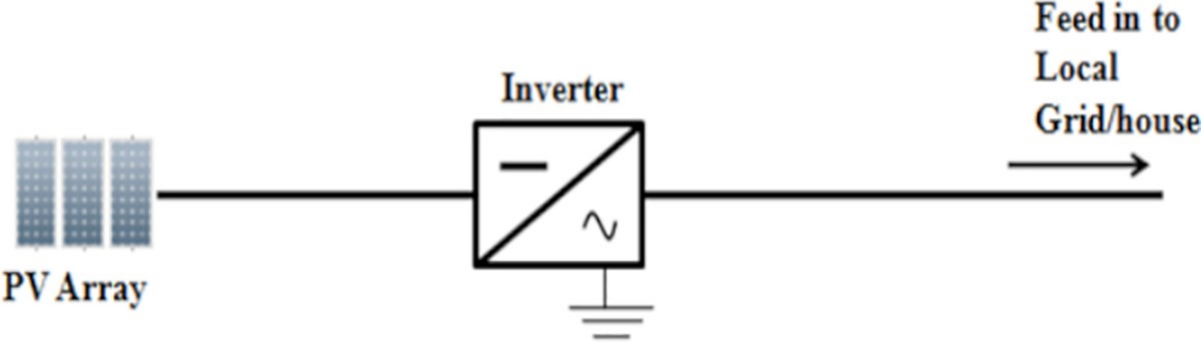
Solar PV rooftop system.

The solar PV Rooftop system with PV array, inverter and grid was modelled in the PSCAD/EMTDC software as presented in Fig 9.

**Fig 10:**
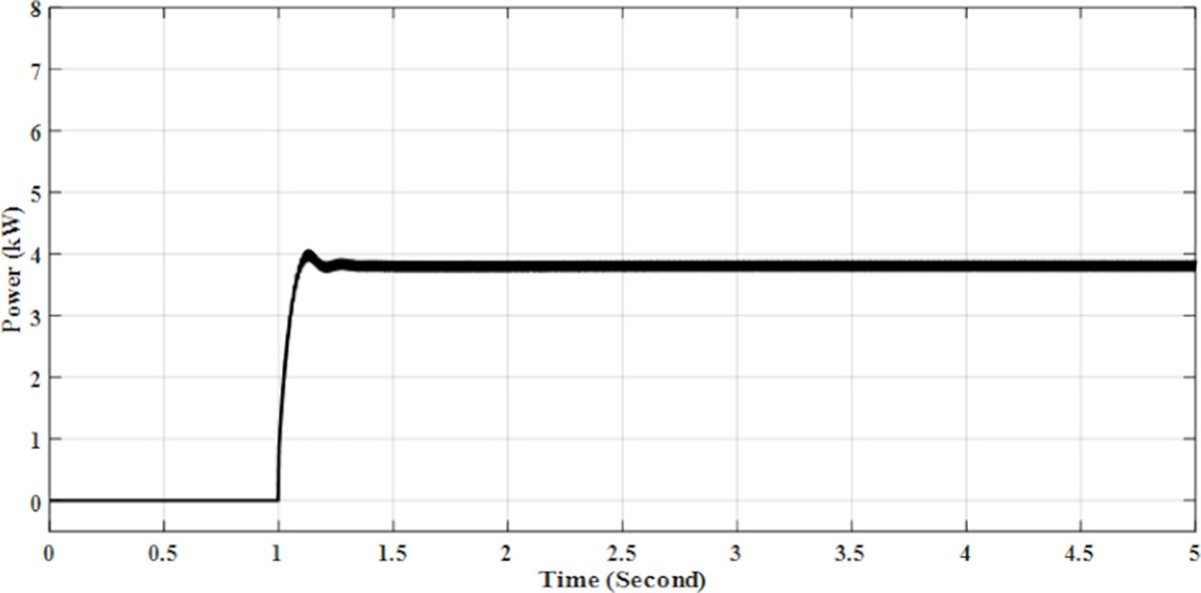
DC input power for solar PV rooftop.

From Table 2, the parameters for Solar PV Rooftop system are constructed from 2 PV arrays, which include a total of 17 modules. The total input power from this system is 3.91 kW at 1000 W/m2 irradiance and a temperature of 25°C at standard test conditions. However, the results in the simulation indicate that the system generates 3.87 kW of energy as shown in Fig 10, and has 98% efficiency. The other 2% of the efficiency is lost due to power losses of the system.

**Table 2:** Parameters for modelling in the PSCAD/EMTDC.

Components	Quantities	Specifications
Solar Modules	17 modules	Power solar PV modules = 3.91 kW
		Voltage of string 1 (8 modules) = 338.4 V
		Current of string 1 = 5.45 A
		Voltage of string 2 (9 modules) = 380.7 V
		Current of string 2 = 5.45 A
Inverter	1	Nominal AC Power = 4 kW
Grid	-	Power Output = 3.91 kW

**Fig 11:**
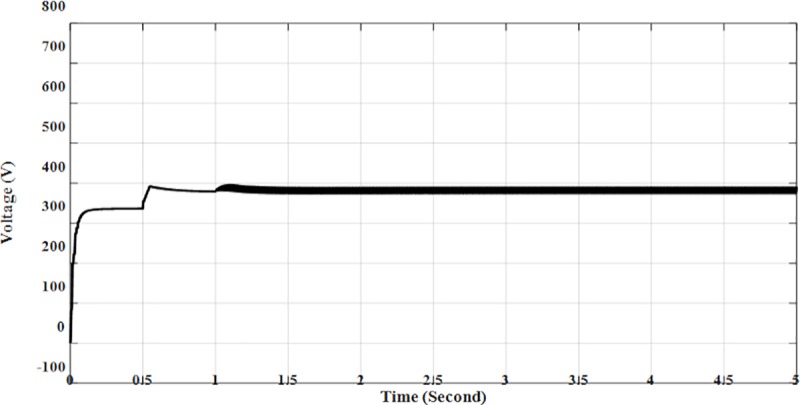
DC voltage for solar PV rooftop.

**Fig 12:**
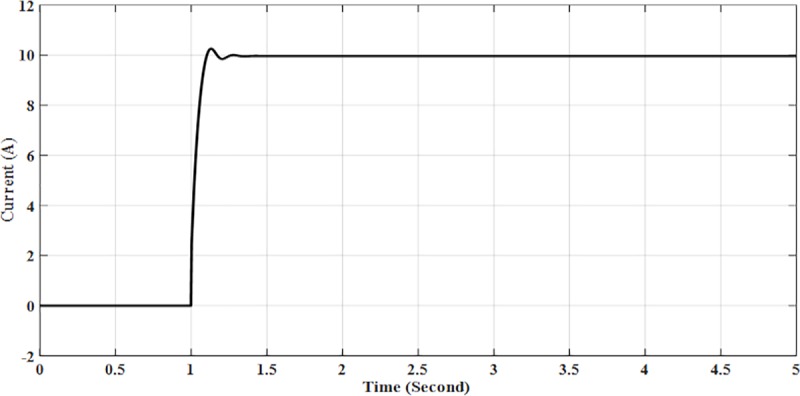
DC current for solar PV rooftop.

**Fig 13:**
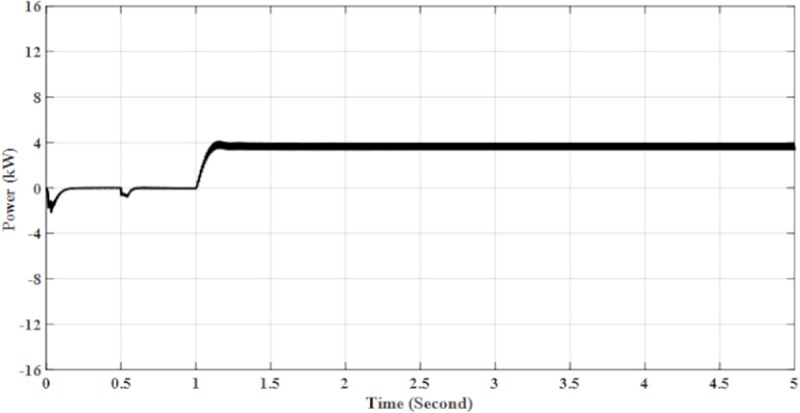
AC output power for solar PV rooftop.

The results of the simulation of Solar PV Rooftop indicate that the DC voltage is 380.1 V as shown in Fig 11, while the DC current is 9.95 A as shown in Fig 12. The AC output power is 3.84 kW as shown in Fig 13.

### Validation of surge protection devices

The Metal oxide surge arrestor in PSCAD/EMTDC is validated with manufacturer’s data. Two types of SPDs type II were validated. namely 500VDC; and 385 VAC. Then, these SPDs were tested using Heidler function with lightning current wave shape of 8/20 μs, which is commonly stated in manufacturer’s technical datasheet.

#### SPD type II 500VDC

From Table 3, manufacturer’s technical data were used to define I-V characteristic to model Metal oxide surge arrestor [24].

**Table 3:** Technical data.

Parameter	Value
*U*_*p*_	20 kA
*I*_*n*_	1.75 kV
*U*_*c*_	500 V

**Fig 14:**
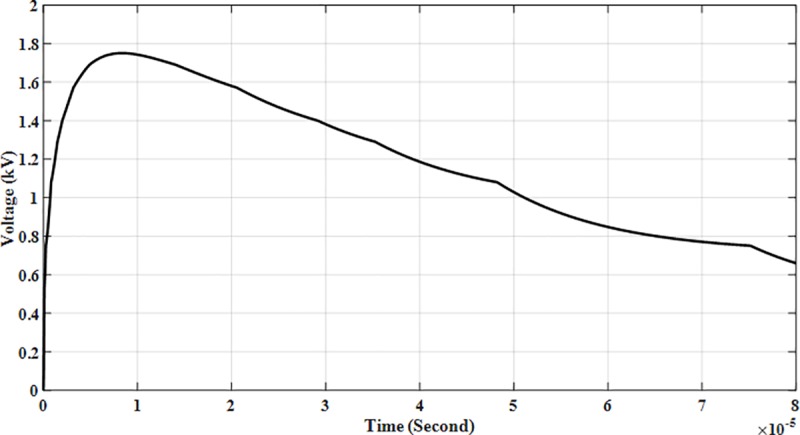
Arrester discharge voltage at *I*_*n*_, 20 kA.

To verify the results, the Heidler function waveform of 8/20 μs was generated and applied to the AC SPD Type II 500 VDC model arrester to acquire satisfactory matching of the discharge voltage. It was tuned based on the data of the manufacturer until the test circuit provided good matches which were: 20 kA produces 1.75 kV for discharge voltage as shown in Fig 14.

#### SPD type II 385 VAC

Likewise for this type of SPD, the technical data as per Table 4 were considered for modelling.

**Table 4:** Technical data.

Parameter	Value
*U*_*p*_	20 kA
*I*_*n*_	1.8 kV
*U*_*c*_	385 V

**Fig 15:**
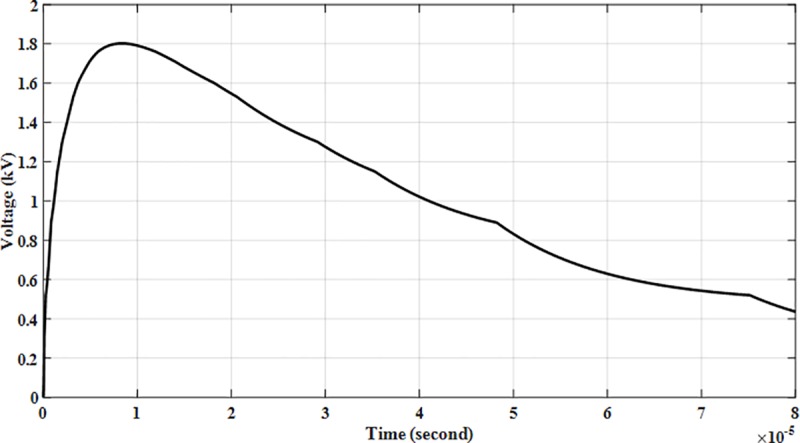
Arrester discharge voltage at *I*_*n*_, 20 kA.

In verifying the results, the Heidler function waveform of 8/20 μs was generated and applied to the AC SPD Type II 385 VACs model arrester to acquire satisfactory matching of the discharge voltage. It was tuned until the test circuit provided good matches based on the data of the manufacturer which were: 20 kA producing 1.8 kV for discharge voltage as shown in Fig 15.

## Results

**Fig 16:**
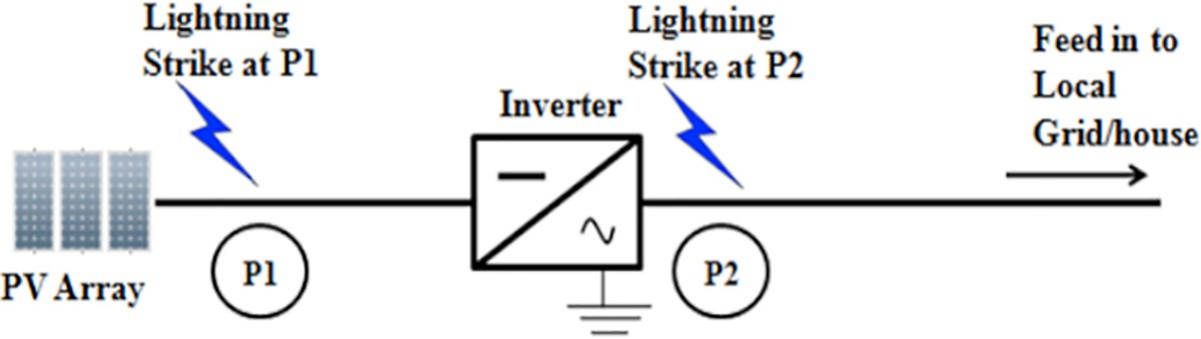
Lightning strikes at the two points of the solar PV rooftop.

This section discusses the effects of the solar PV Rooftop system installation with the inclusion SPD and without the inclusion of SPD as there are no specific practices that focus on it. Lightning strike with different striking points, different lightning impulse current wave shapes, and variation of amplitude of currents, different cable lengths and sizes are considered in this section of the work. The two points are seen between: the inverter and the solar PV; array (P1); and the inverter and grid (P2) as presented in Fig 16.

**Fig 17:**
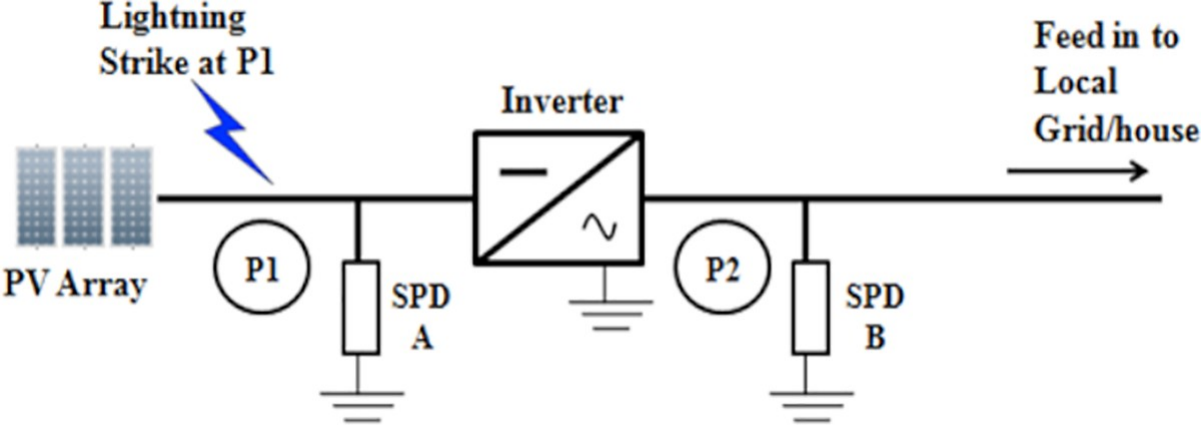
Lightning strike at point 1 between PV array and inverter.

**Fig 18:**
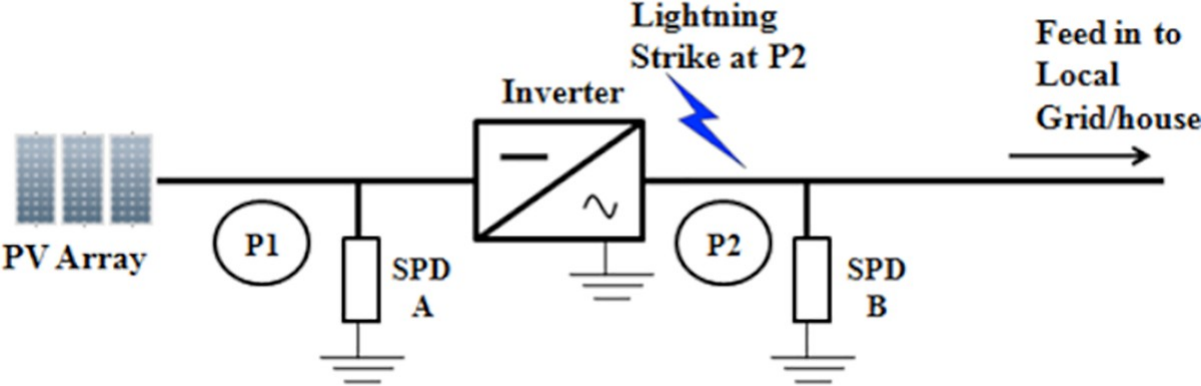
Lightning strike at point 2 after inverter.

SPD is an essential part that is most commonly used for overvoltage protection at solar PV Rooftop or PV farm system due to its efficiency. Therefore, all the SPD installations at the DC or AC part must meet the requirement or practice from the standard. Figs 17 and 18; indicate that lightning will strike at P1 and P2 of the solar PV Rooftop system.

As per the standard, SPD Type II installation uses the lightning impulse current waveshape of 8/20μs. Due to direct lightning strike on certain points of the PV Rooftop system, extremely high current and voltage propagated as travelling waves are produced. Therefore, several case studies need to be considered to minimise the transient current and voltage to avoid the damage or decaying of electronic and electrical equipment. Furthermore, this section will consider the SPD rating and placement. Then, the SPD model applied in this simulation is validated accordingly to follow the manufacturer’s datasheet. The significance of the simulation outcomes of the solar PV Rooftop system under the influence of lightning is seen in this study.

### Impact of different points of strikes

This study investigates the effect on the components when lightning strikes at two different points of the installation. These two points can be seen between the inverter and the solar PV array (**P1**) and between inverter and grid (P2) as shown in Fig 16. The findings of the lightning strike at P1 and P2 are then observed. The parameters used are: 31 kA peak current; 10 metre-length of cable; and the lightning impulse current waveshape, 8/20μs. Results of these cases, are tabulated in Tables 5 and 6.

**Table 5:** Measured Vinv and Iinv at striking point.

	Without Surge Protection Device (SPD)				With Surge Protection Device (SPD)			
Lightning Amplitude (kA)	P1 (before Inverter)		P1 (After Inverter)		P1 (before Inverter)		P1 (After Inverter)	
	Vinv DC	Iinv DC	Vinv AC	Iinv AC	Vinv DC	Iinv DC	Vinv AC	Iinv AC
	kV	kA	kV	kA	kV	kA	V	kA
31	2397	31	5392	5.97	1.915	0	0.751	0

**Table 6:** Measured Vinv and Iinv at P2 striking point.

	Without Surge Protection Device (SPD)				With Surge Protection Device (SPD)			
Lightning Amplitude (kA)	P2 (before Inverter)		P2 (After Inverter)		P2 (before Inverter)		P2 (After Inverter)	
	Vinv DC	Iinv DC	Vinv AC	Iinv AC	Vinv DC	Iinv DC	Vinv AC	Iinv AC
	kV	kA	kV	kA	mV	kA	kV	kA
31	0.153	0	4283	25.353	0.01116	0	2.033	0.292

Extremely high current and voltage which are propagated as travelling waves are produced due to the direct lightning strike on certain points of the PV Rooftop system. Modules of solar PV tend to be damaged and degrade due to the lightning strikes at P1 and P2. Referring to the data, the findings suggest that electrical and electronic equipment is most susceptible to damage.

From the data in Table 4, it is apparent that the high current and voltage at P1 striking point are 31 kA and 2397 kV, respectively. As for the AC part the current and voltage values were found to be 5.97 kA and 5392 kV, respectively. From Tables 5 and 6, the lightning waveshape 8/20 μs will strike at P1 and P2. Then, from Tables 5 and 6, the results show that high transient current voltage can be expected to clamp sharply at the values in kV and mA.

### Impact of lightning impulse current waveshapes

**Fig 19:**
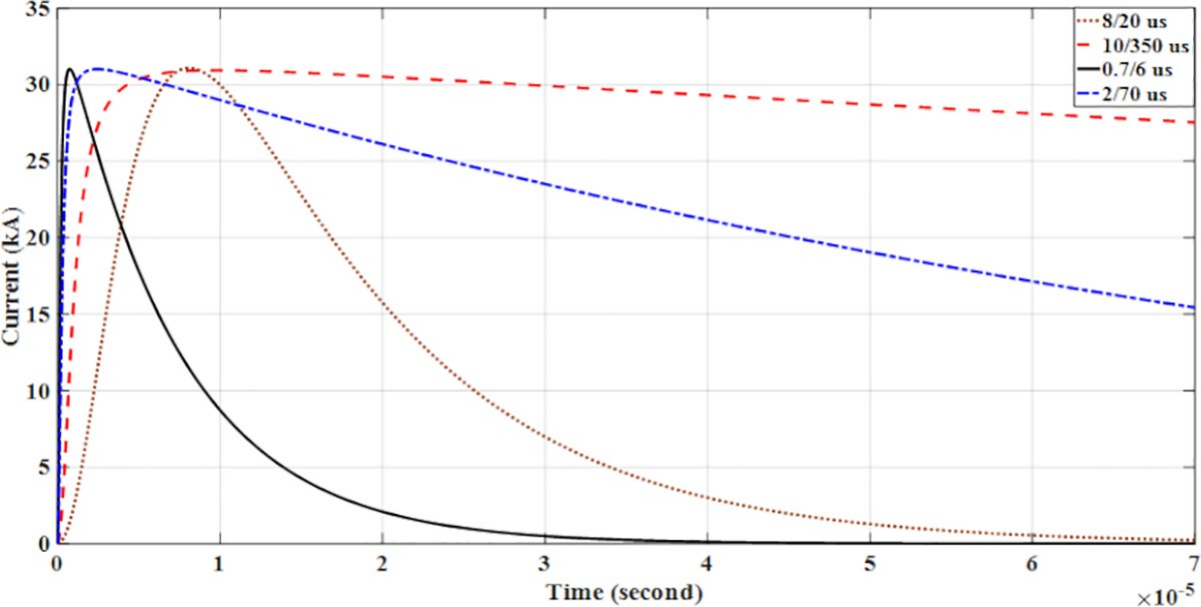
Variation of lightning impulse current wave shape.

Four variations of lightning impulse current wave shapes: 10/350μs; 8/20μs;0.7/6μs;and 2/70μs are discussed with regard to their impacts in this section. Variations of lightning impulse current wave shapes are applied at P1 and P2 to study their effects on the solar PV Rooftop. From Tables 6 and 7, all the data were measured by applying the four different lightning impulse current wave shapes at P1 and P2 with the same peak of lightning amplitude of 31 kA as shown in Fig 19.

**Table 7:** Variation of lightning impulse current wave shape at P1 striking point.

	Without Surge Protection Device (SPD)				With Surge Protection Device (SPD)			
Lightning Impulse Current Wave shape at, μs	P1 (before Inverter)		P1 (After Inverter)		P1 (before Inverter)		P1 (After Inverter)	
	Vinv DC	Iinv DC	Vinv AC	Iinv AC	Vinv DC	Iinv DC	Vinv AC	Iinv AC
	kV	kA	kV	kA	kV	mA	V	kA
8/20	1915	31	5392	5.97	1.915	21.10	0.751	0
10/350	2479	31	11865	6.618	1.913	33.58	0.897	0
0.7/6	2392	31	3053	10.987	1.914	15.53	0.614	0
2/70	2391	31	9887	9.885	2.391	31.53	0.867	0

Further analysis in Tables 7 and 8 shows that when lightning strikes at P1 and P2, the highest voltage is obtained at wave shape of 10/350 μs, followed by 2/70 μs, 8/20 μs and 0.7/6 μs. Therefore, the solar PV Rooftop modules and inverter have a tendency to decay and be damaged due to the current waveshape applied.

**Table 8:** Variation of lightning impulse current wave shape at P2 striking point.

	Without Surge Protection Device (SPD)				With Surge Protection Device (SPD)			
Lightning Impulse Current Wave shape at, μs	P2 (before Inverter)		P2 (After Inverter)		P2 (before Inverter)		P2 (After Inverter)	
	Vinv DC	Iinv DC	Vinv AC	Iinv AC	Vinv DC	Iinv DC	Vinv AC	Iinv AC
	kV	kA	kV	kA	mV	A	kV	kA
8/20	0.153	0	4283	25.353	0.01116	0	2.033	0.292
10/350	0.203	0	11277	26.003	0.01289	0	2.029	0.592
0.7/6	0.0911	0	1924	26.309	0.01074	0	2.033	0.144
2/70	0.193	0	8773	26.305	0.01337	0	2.032	0.537

Another important finding as shown in Table 7 is that transient voltage becomes slightly different. However, among the four lightning impulse current wave shapes, the 10/350μs shows high value of transient current of 33.58 mA was clamped. The table shows that 2/70μs impulse current wave shape has high value of transient voltage that was also clamped at 2.391 kV for lightning strike at P1. At high transient current, 0.592 kA, P2 was clamped for lightning impulse current wave shape at 10/350μs.

### Impact of lightning current amplitude

Impact of different lightning amplitude current is described in this section. The parameters that are used in this case study include: the 31 kA peak current; 10 metres length of cable; and the lightning impulse current waveshape,8/20μs. Different lightning amplitudes were also applied, ranging from 2–200 kA selected based on the CIGRE distribution. The currents were injected at P1 and P2 at the solar PV Rooftop system and data were measured as shown in Tables 9 and 10.

**Table 9:** Impact of variation lightning amplitude current at P1 striking point.

	Without Surge Protection Device (SPD)				With Surge Protection Device (SPD)			
Lightning Amplitude (kA)	P1(before Inverter)		P1 (After Inverter)		P1 (before Inverter)		P1 (After Inverter)	
	Vinv DC	Iinv DC	Vinv AC	Iinv AC	Vinv DC	Iinv DC	Vinv AC	Iinv AC
	kV	kA	kV	kA	kV	mA	V	kA
2	154	2	348	0.385	1.185	13.42	0.596	0
5	386	5	869	0.964	1.3999	15.40	0.645	0
10	773	10	1740	1.929	1.571	17.62	0.679	0
20	1549	20	3487	3.866	1.751	19.88	0.719	0
30	2326	30	5235	5.803	1.902	21.02	0.748	0
40	3085	40	6943	7.697	2.049	22.03	0.774	0
50	3863	50	8695	9.639	2.199	23.01	0.796	0
100	7719	100	17373	19.260	2.948	27.36	0.897	0
150	11570	150	26041	28.869	3.694	31.15	0.989	0
200	15428	200	34723	38.495	4.443	36.03	1.08	0

**Table 10:** Impact of variation lightning amplitude current at P2 striking point.

	Without Surge Protection Device (SPD)				With Surge Protection Device (SPD)			
Lightning Amplitude (kA)	P2(before Inverter)		P2 (After Inverter)		P2 (before Inverter)		P2 (After Inverter)	
	Vinv DC	Iinv DC	Vinv AC	Iinv AC	Vinv DC	Iinv DC	Vinv AC	Iinv AC
	kV	kA	kV	kA	mV	kA	kV	kA
2	0.00988	0	276	1.634	0.004431	0	1.015	0.0823
5	0.0247	0	691	4.089	0.006211	0	1.297	0.131
10	0.0495	0	1381	8.178	0.007772	0	1.528	0.181
20	0.0991	0	2769	16.392	0.009703	0	1.802	0.244
30	0.149	0	4156	24.607	0.01105	0	2.013	0.288
40	0.198	0	5522	32.697	0.01228	0	2.222	0.325
50	0.247	0	6904	40.875	0.01348	0	2.432	0.359
100	0.494	0	13794	81.670	0.01934	0	3.483	0.502
150	0.740	0	26675	122.414	0.02512	0	4.532	0.629
200	0.987	0	27569	163.231	0.03089	0	5.583	0.750

The electrical and electronic equipment has the highest tendency to be damaged or to decay even at 2 kA of lightning amplitude current strike at P1 and P2. The current at 2 kA lightning amplitude strike is considered high while having voltage values of 154 kV at P1 and 276 kV at P2.

Tables 9 and 10 show the case of Variation Lightning Amplitude Current, 2 kA to 200 kA with type II SPD Installation at DC and AC side. The tables show that there is a slight increase in the value of transient current and voltage clamped based on the observation done from the table. There is a high possibility that the highest transient current and voltage at lightning amplitude current of 200 kA would be clamped from 15.428 MV to 4.443 kV for lightning strikes at P1 and 34.723 MV to 1.08 V for lightning strike at P2.

### Impact of cable lengths

The impact of lightning on varying cable lengths at P1 and P2 are discussed in this section. The parameters used in this part of the study are: 31 kA peak current; and lightning impulse current waveshape, 8/20μs. Different cable lengths from 5 metres to 20 metres are applied to the parametres accordingly at point 1 and point 2. From Tables 11 and 12, all the data measured will be discussed in relation to the impact of cable length variation on the PV Rooftop system.

**Table 11:** Variation of cable length at P1.

	Without Surge Protection Device (SPD)				With Surge Protection Device (SPD)			
Cable Distance (m)	P1 (before Inverter)		P1 (After Inverter)		P1 (before Inverter)		P1 (After Inverter)	
	Vinv DC	Iinv DC	Vinv AC	Iinv AC	Vinv DC	Iinv DC	Vinv AC	Iinv AC
	kV	kA	kV	kA	kV	mA	V	kA
5	2397	31	7579	4.005	1.915	56.24	1.17	0
10	2397	31	5392	5.97	1.915	21.10	0.751	0
15	2397	31	4447	7.444	1.915	16.75	0.603	0
20	2397	31	3834	8.672	1.915	15.53	0.566	0

**Table 12:** Variation of cable length at P2.

	Without Surge Protection Device (SPD)				With Surge Protection Device (SPD)			
Cable Distance (m)	P2(before Inverter)		P2 (After Inverter)		P2 (before Inverter)		P2 (After Inverter)	
	Vinv DC	Iinv DC	Vinv AC	Iinv AC	Vinv DC	Iinv DC	Vinv AC	Iinv AC
	kV	kA	kV	kA	mV	kA	kV	kA
5	0.169	0	6448	25.357	0.07406	0	2.032	0.348
10	0.153	0	4283	25.353	0.01116	0	2.033	0.292
15	0.00237	0	3152	25.103	0.0000308	0	2.033	0.251
20	0	0	2286	24.786	0.000002933	0	2.033	0.221

In summary, the results in Table 11 show that the current and voltage at DC part are the same when lightning strikes at P1 and at AC part the results indicate that the highest voltage and current is at the 5-metre cable length. When lightning strikes at P2 the results indicate that the highest voltage and current is at the 5-metre cable length.

The next section of the results pertains to different cable lengths as shown in Table 11. The transient current at P1 before the inverter for a 5-metre cable length is high compared to the other lengths of 56.24 mA. The transient current decreases as the cable length increases until the length of 20 metres. However, the transient current and voltage clamped among the four cable lengths at P1 and P2 are almost the same.

## Discussion

The investigations of lightning strikes in several case studies were conducted and their outcomes discussed. When lightning directly strikes at certain points of the PV Rooftop system, extremely high voltage and current that exceed the acceptance level of voltage are produced. In this section, according to the lightning amplitude at 31 kA peak with 8/20 μs lightning impulse current, the high current and voltage at P1 striking point were 31 kA and 2397 kV, respectively. As for the AC part of this section, the current and voltage values were found to be 5.97 kA and 5392 kV, respectively. Without suitable protection, equipment is susceptible to failure and damage from the high voltages and currents. Therefore, SPDs with suitable ratings provided by SEDA were deployed. Results also show that high transient current voltage is expected to clamp sharply at the values of 1.915 kV and 0 ampere at the P1 striking point. The AC part shows that the current and voltage values were 0 kA and 0.751 V, respectively. The rising time and decaying time of lightning current wave shape will affect the transient voltage as shown. Moreover, if rise time and decay time are increased, the transient voltage increases too.

Direct lightning strikes by lightning current impulse wave shape of 10/350 μs with a fast rise time and long decaying time produce high energy. The high current and voltage that were produced at the P1 striking point were 31 kA and 2479 kV for this impulse wave shape. In the AC part the current and voltage values were found to be 6.618 kA and 11865 kV, respectively. High values of transient current and voltage were clamped at a lower level at values of 33.58 mA and 1.913 kV. The AC part shows that the current and voltage were 0 ampere and 0.897 volt. In this section, the impact of varying cable lengths on the PV Rooftop system shows that for the AC part, the highest voltage and current was at the 5-metre cable length. Consequently, according to the results, there is still the need to deploy SPD at points 1 and 2 as opposed to the standard at the 5-metre length of cable.

To ensure that there is effective protection of the equipment, the value of Voltage protection level, U_p_ should be lower than the value of voltage withstand of the equipment to be protected. A safety margin of at least 20% between the voltage withstand of the equipment and U_p_ should be maintained. Results obtained have given some indicators on the proper ratings to be made depending on the local data available at the site. Parameters like MCOV, Up, Imax and Iimp play an important role when selecting the right SPD to be installed. This does not only help in protecting the system from damage but will increase the life span of the components in the long run.

## Conclusion

Due to its spacious structure and is full exposure outdoors, PV Rooftop systems are highly prone to direct and indirect lightning strikes and other surge overvoltages. These disruptions may cause destruction and malfunction of part of the whole PV Rooftop system. Direct strikes lead to severe damage to the PV Rooftop system. A lightning strike nearby induces voltages into the system. These strikes can also destroy PV panels, inverters, cables, and fuses that consequently disrupt the operation of the system. The Lightning Protection technique for PV Rooftop system should be designed properly and implemented for economic reasons and ensure return of investment. Therefore, rather than incurring the high cost of repair and replacement due to damage from lightning strikes, it is prudent to provide a safe location to install and optimise the lightning protection of the PV Rooftop System.

From the analyses done without the inclusion of SPDs, modules of solar PV would be damaged and degraded if they exceed their withstanding capabilities. The extremely high current and voltage that are propagated as travelling waves produced by a direct lightning strike on certain points of the PV Rooftop system are responsible for this. In the long run, lightning tends to damage or degrade the electronic equipment of the PV Rooftop system. Regardless of any point of strike, there is a need for SPD to be installed. Results obtained have given some indications of the proper ratings to be made, depending on the local data available on site. The criteria when selecting the right SPD to be installed are also important. This does not only assist in protecting the system from severe damage but will increase the life span of the components and generally ensure the smooth and efficient operation of the PV Rooftop system.
